# Analysis of the maize and groundnut seed value chains in central and northern Tanzania

**DOI:** 10.1186/s40066-026-00623-x

**Published:** 2026-10-02

**Authors:** MacDonald B. Jumbo, James Mwololo, Julius Manda, Philip Kamau, Wills Munthali, Patrick Okori, Dan Makumbi, Hapson Mushoriwa, Irmgard Zeledon, M. Bekunda, Walter Chivasa, BM Prasanna

**Affiliations:** 1 International Crops Research Institute for the Semi-Arid Tropics (ICRISAT), Matopos Research Station, P.O. Box 776, Bulawayo, Zimbabwe; 2 International Crops Research Institute for the Semi-Arid Tropics (ICRISAT), Chitedze Agricultural Research Station, P.O. Box 1096, Lilongwe, Malawi; 3 International Institute of Tropical Agriculture (IITA), AVRDC- The World Vegetable Centre, P.O. Box 10, Duluti, Arusha Tanzania; 4 International Institute of Tropical Agriculture (IITA), PMB 5320, Oyo Road, Ibadan, 200001 Oyo State Nigeria; 5 International Maize and Wheat Improvement Center (CIMMYT), Village Market 00621, P.O. Box 1041, Nairobi, Kenya

**Keywords:** Value chain analysis, Maize seed, Groundnut seed, Quality Declared Seed, Value chain actors, Community seed banks, Tanzania

## Abstract

**Background:**

Maize and groundnut are important crops in Tanzania; therefore, understanding their seed value chains (VCs) is critical for improving the seed systems. The study aimed at establishing key components for model maize and groundnut seed VCs by understanding the formal and informal seed VCs to provide relevant recommendations for; improving these value chains, propose interventions that would encourage more investment by seed companies, and improve access to quality seed by farmers. The study used data collected through survey, with a sample size of 252 and 162 seed producing households for maize and groundnut, respectively, conducted using structured questionnaire. VC actors including; seed companies, agricultural research institutions, agrodealers, seed regulatory authority, public seed production agency, extension staff and Ministry of Agriculture, were also interviewed. *Porter’s value chain* approach was applied to analyse the various components of the VC and identify the links between the key nodes and understand their operational functions.

**Results:**

The maize seed producing households were dominated by men (71%) while for groundnut, women (69%) were engaged in seed production. Government agricultural extension services are the main source of information; 30% and 58%, for maize and groundnut, respectively. Seed companies were the main producers of maize seed (51.7%) while for groundnut, public research institutions were the main seed producers. Most of business enterprises were owned by men (83.3%). Compared to maize, the formal seed sector is weak for groundnut in Tanzania, with very few private seed companies being engaged in seed production and supply. Most groundnut seeds are accessed through agrodealers and seed associations that produce quality declared seed (QDS). Community seed banks were recognized as an efficient option for providing improved groundnut varieties to smallholder farmers. There are no prominent seed companies aggressively operating for both crops in central Tanzania. Excessive reliance on farm-saved seed by farmers was identified as one of the major factors negatively affecting the demand for certified seed. Stakeholder analysis of the seed production and supply chain showed that there was poor coordination between key stakeholders involved in the seed VC.

**Conclusion:**

This study provides recommendations for strengthening the seed value chains in Tanzania by addressing identified gaps along the VC components. Future studies can build from the outcomes of this study and generate more information, especially on market and different seed systems, as these have great influence on demand and supply of seed.

## Introduction

 In Tanzania, maize and groundnut are major staple food crops, with maize annual production estimated at 6 million tons; accounting for 70% of cereals produced, while groundnut production was 690,000 tons [1, 2]. Surplus grain of maize and groundnut is usually sold and contributes significantly as a source of income for most households. Maize and groundnut residue are used as fodder for livestock.

Groundnut is produced in most areas below 1500 m above sea level [3], and its production has increased in recent years but is still low compared to maize. After sunflower, groundnut ranks second in oilseed production in Tanzania [4]. Despite their importance, the productivity of maize and groundnut in Tanzania is still low (1.4tha^− 1^ and 0.7t ha^− 1^, respectively) [1]; due to many factors, major ones being biotic and abiotic stresses. In Kongwa and Kiteto districts, maize and groundnut production face several challenges, including harsh weather conditions, poor soil fertility, emerging pests (Fall Armyworm), and diseases (Maize Lethal Necrosis; groundnut rosette). Several varieties have been developed and released in Tanzania to address these problems, but their performance in drier regions still has some challenges.

Evaluating promising maize and groundnut varieties using the more representative semi-arid environments is the best approach for identifying and recommending superior varieties for release in marginal areas. Based on this approach, several new maize and groundnut varieties were evaluated by the International Maize and Wheat Improvement Center (CIMMYT) and International Crops Research Institute for the Semi-Arid Tropics (ICRISAT) in Kongwa and Kiteto districts for yield and agronomic performance. This led to the release of the two new best-performing, drought-tolerant, quality protein maize hybrids in the target region, while three groundnut varieties were proposed for release.

Although more new varieties with better tolerance to biotic and abiotic stresses are being released, the low rate of commercialization and lack of incentives to invest in high-risk environments by seed businesses has left farmers in the drier regions with limited variety options, availability, and access to quality seed of improved varieties. In Tanzania, most of the local and foreign seed companies tend to concentrate the seed production and marketing business in the high potential regions of the Southern highlands (Rukwa, Mbeya, and Iringa) and Northern highlands (Manyara, Kilimanjaro, and Arusha), reducing the opportunity for farmers in semi-arid areas accessing quality seed of improved varieties.

Efforts to increase access to farm inputs by different stakeholders, including government, local and international Nongovernmental Organizations (NGOs), are on the rise; however, adoption (replacement and diffusion) of new crop varieties remains a persistent challenge among smallholder farmers [5,7]. In drier areas, adopting improved drought-tolerant maize varieties remains low in Tanzania despite huge investments made in variety development by public and private sector researchers [8]. Similarly, the cultivation of old groundnut varieties dominates, with very few farmers accessing the new improved varieties.

Limited resource endowment and risk aversion are some of the major factors attributed to the low adoption of new crop varieties [7, 9]. Risk aversion also contributes to some smallholder farmers’ failure to choose hybrids over the local maize varieties, mainly due to unpredictable climate vagaries. Farmers perception is that recycled local varieties have minimal risk in case of crop failure, however, the yields of these varieties are significantly lower compared to hybrids. On the other hand, the diffusion of new groundnut varieties is largely impeded by lack of investment by the private sector in the seed value chain, similar to many other legume crops. The major focus is on the maize sub-sector while other seed sub-sectors, such as legumes, including groundnut, that are equally important for household livelihoods, remain underdeveloped. Most smallholder farmers in Tanzania rely on the informal seed system, especially for underinvested crops such as legumes, due to factors that include limited resources to purchase seed, and poor access to agrodealers [4]. Quality declared seed (QDS), which is recognized in Tanzania, is a good option for producing quality seed. However, standards or frameworks for seed inspection and regulations to support QDS production are still not well established. This study analysed factors related to the challenges mentioned above. It explored possible options as recommendations to them using lessons and successes from a formal seed system compared to the informal seed system of the seed value chains. Considering the importance of maize and groundnut in Tanzania, there is a need to understand the formal and informal seed value chains to: (i) draw relevant recommendations for improving these value chains, (ii) propose interventions that would encourage more investment by seed companies, and (iii) stimulate adoption of new improved varieties by farmers. The seed value chain analysis (VCA) for both maize and groundnut is therefore critical for the stakeholders to understand issues that lead to underinvestment, thus affecting access to seed of improved varieties in the drier areas.

Unlike many value chain studies [10, 11] that have concentrated on grains, we specifically examine the seed value chains in this study. We use a comprehensive data set from actors in the maize and groundnut value chains to assess the critical factors that influence the seed value chain of maize and groundnut and to establish the key elements of a “well-designed seed value chain development initiative”. By so doing, the study seeks to provide evidence on how the formal and well-coordinated seed VC operates (the case of hybrid maize seed) and adapts critical successes to improve the formal and informal seed VC of legume seed (the case of groundnut) and open-pollinated maize. In addition, the study explored the challenges of deploying new improved varieties in dry areas, such as the case of central Tanzania.

### Conceptual framework

The value chain denotes a set of linked economic agents who perform a group of dependent economic activities [12]. The economic agents (actors) perform a set of combined activities to produce a particular output (end product). Along the chain, value is added to a product such that the downstream players become customers to the upstream players. VCA is used to get insights into the evolution of activities and processes required to bring agricultural products/services from conception through production, delivery, and end-use by consumers [13]. Value chain analysis aims to ascertain upgrading approaches of the chain to ensure consistency and stability in delivering quality and quantity of product/service [14]. The current study used one of the enterprise value chain approaches known as *Porter’s value chain*, which is considered as the consumer-driven and collaborative value chain approach [15, 16].

## Methodology

### Description of the study area

**Fig. 1:**
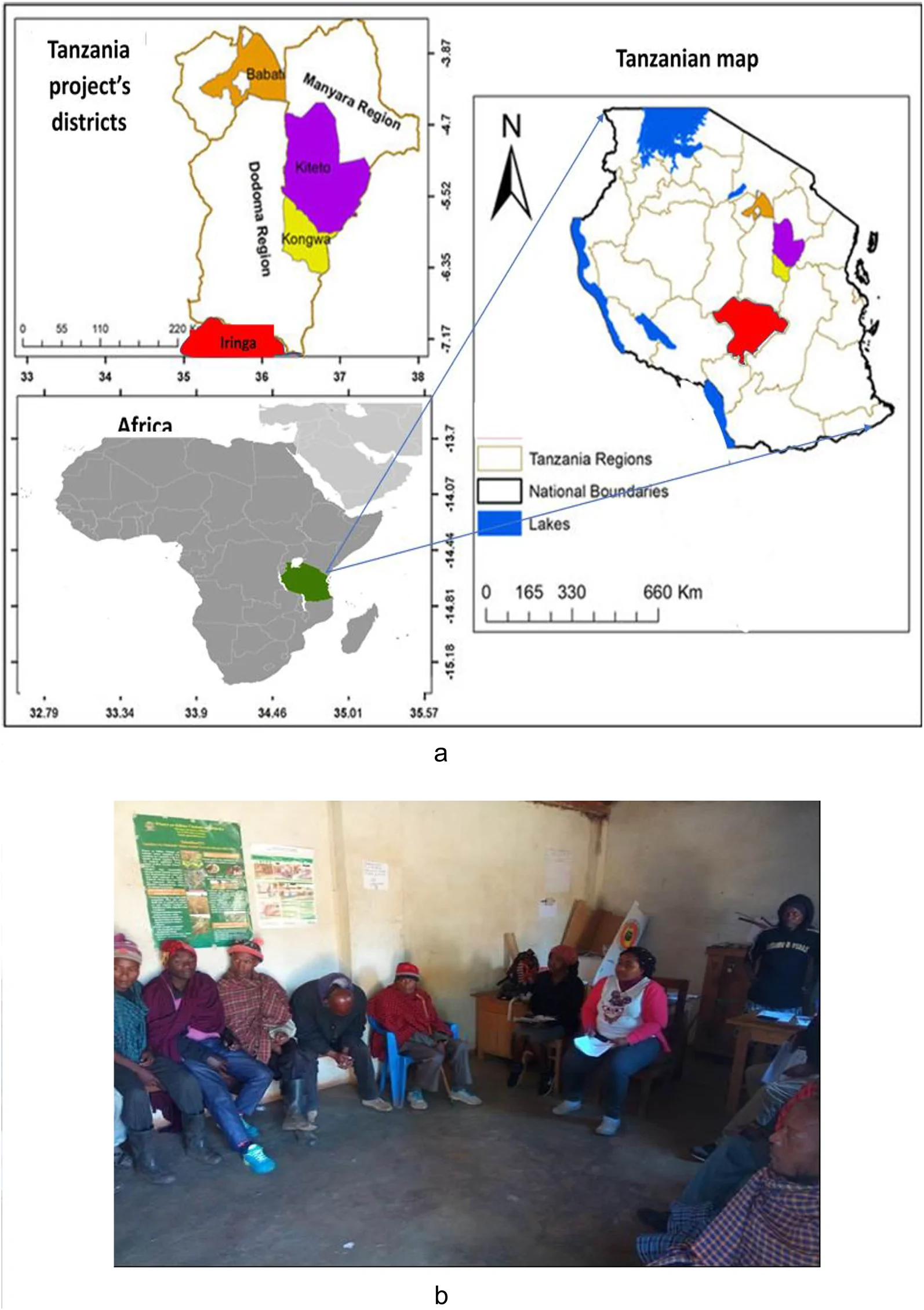
**a**) Map of Tanzania showing Dodoma, Kongwa, Kiteto, Iringa and Babati **b)**. A session of focused group discussion with farmers

The study was conducted in Kongwa (Dodoma region), Kiteto and Babati (Manyara region), and Iringa district (Iringa region). While Kongwa, Kiteto and Iringa have semi-arid climatic conditions, Babati district has optimal climatic conditions and is well-accessed by the formal seed sector, particularly on maize. Therefore, Babati was selected for comparison with the aforementioned districts in the two semi-arid regions (Fig. 1a).

The study considered five different agroecological zones (AEZ), namely; Humid highlands, Sub-humid highlands, Semi-humid uplands and Semi-humid (zones which are considered as high potential áreas receiving optimal rainfall between 1,000 mm to 2000 mm per annum or more), and Semi-arid midlands (considered as drier areas with less seed business investment; receiving below 1000 mm of rainfall per annum). The villages for each zone included Gawal in the Humid highlands, Secheda in the Semi-humid uplands, Duru in the Sub-humid highlands, Gedarmar and Ismani for the Semi-humid midlands, and Mlali, Kiperesa, and Ng’humbi for the Semi-arid Midlands (Table 1).

**Table 1:** Number of respondents in Kongwa, Kiteto, Iringa and Babati districts

		Maize		Groundnut								
District	Village	^±^HH	Number of FDG^†^	HH^**±**^	FDG^†^	SD^$^	^≠^TD	^±±^Agd	^‡^RI	^††^D. Ext Off	^‡‡^VG Ext Off	TOSCI
						5	7	29	4		4	4
Kongwa	Ng’humbi	32	1	41	20					1		
Kongwa	Mlali	33	1	45								
Kiteto	Kiperesa	31	1	37	20							
Iringa	Ismani	30	1	39						1		
Babati	Secheda	32	1	0						1		
Babati	Gawal	31	1	0								
Babati	Duru	33	1	0								
Babati	Gedarmar	30	1	0								
Total		252	8	162	40	5	7	29	4	3	4	4

^**±**^Households, ^†^Focus groups, ^≠^Traders, ^±±^Agrodealers, ^**±**^Focus Discussion Groups, ^‡^Research Institute, ^‡‡^Village Extension Agents, ^$^Seed Companies, ^††^District Extension Officer

The baseline district for hybrid maize production was Babati, where four villages were purposively selected in each of the AEZ due to strong access to input and output markets. They are also easily accessed by the extension agencies and the Quality Declared Seeds (QDS) inspectors. This is a high potential area where hybrid maize production is key, used for comparison considering that the maize seed value chain has more private sector participation (commercial seed companies) which is mostly active in these high potential areas while the groundnut seed value chain has very limited private sector participation and predominant in the low potential areas that include the districts of focus; which are characterised by water stress.

### Sampling and data collection

Data were collected from primary sources using structured questionnaires which were administered to the following value chain actors (VCA) (Tables 1 and 2); seed producer households, commercial seed producers and suppliers (Seed companies), business agents (agrodealers), Research Institutions (Tanzania Agricultural Research Institute; TARI), Seed Certification Authority (Tanzania Official Seed Certification Institute; TOSCI), extension services, public seed production unit (Tanzania Agricultural Seed Agency; ASA) and ministry of agriculture (MoA). The description and roles of the actors are presented in Table 2.

**Table 2:** Description and roles of the maize and groundnut seed value chain actors in Tanzania

Value chain actor	Description and role
Seed producer households	Smallholder farmers that specialize in producing seeds; often of improved or certified varieties for sale or distribution to other farmers, seed companies, or government agencies.
Seed companies	Specialize in producing, processing, marketing, and distributing seeds for agriculture use. They provide farmers with high-quality, genetically pure, high-yielding, drought and pest tolerant seeds.
Agrodealers	Sell agricultural inputs, including seed and services to farmers. They act as a vital link between seed companies and the farmers who use the seed
Tanzania Agricultural Research Institute	TARI is the national body responsible for conducting, coordinating, and promoting agricultural research in Tanzania. It develops new crop varieties, farming techniques, and technologies to improve productivity, sustainability, and resilience in the agricultural sector.
Tanzania Official Seed Certification Institute	Government agency responsible for regulating, certifying, and ensuring the quality of seeds in Tanzania
Extension services	Provide farmers and other stakeholders in the agriculture sector with practical information, training, and support to improve their farming practices, productivity, and livelihoods
Tanzania Agricultural Seed Agency	A government agency under the Ministry of Agriculture, tasked with ensuring the availability, accessibility, and affordability of quality crop seeds for farmers in Tanzania.
Ministry of Agriculture	Government body responsible for formulating, implementing, and overseeing policies, strategies, and programs related to agriculture in the country

Data were also collected through focus group discussions (FGD) (Fig. 1b). The FGDs were composed of maize and groundnut farmers, village executive officers, government extension officers, village headmen, and other civil servants (e.g. agriculture officers who are not extension officers), and community members who are knowledgeable about farming. Included is photo of the FGD session at Gawal Village (Fig. 1b). Using probability proportional to size sampling approach [17], seed producer households in each village, data was collected from 12 selected villages, namely; Mlali, Manyusi, Laikala, Moleti (Kongwa district), Njoro, Kiperesa (Kiteto district), Ismani (Iringa district), Gawal, Secheda, Duru, and Gedarmar (Babati district). Second, households were selected randomly, leading to a sample size of 252 households for maize and 162 households for groundnut. Further, these respondents were selected in consideration of their engagement in farming, their active participation in agricultural activities, some different roles they take in their communities related to agricultural activities. The selection of the respondents was then refined by stepwise selection approach on regression analysis integrating key factors such as sex of the maize farmer, age of the maize farmer, number of years of schooling, number of members living in this household and main source of this information. We accessed these households with the help of village elders and in case the household was not accessible three times, we selected another one from the replacement households. The selected villages in Kongwa, Kiteto and Iringa districts represented the semi-arid region, while the villages in Babati district represented high potential environments.

### Focused group discussion approach

The participants were first asked if they had any questions or clarification regarding the study because they had already been informed about the objective of the study during the listing and sampling exercises. An informed consent form was then read to them, and they all consented to participate in the study. The venue of the FGDs meetings was at the area chief’s premises where proximity factor was considered, and most of the farmers had clear knowledge of the venue. The supervisor sat in the FGDs, not just listening but also correcting if a certain question/section was not correctly asked. Mistakes noted were discussed immediately as the team travelled to the next village. A research assistant was moderating the session to ensure that the discussions were on track, and a note taker was recording the details of the whole exercise. Different topics discussed included maize and groundnut production, sources of seed and other farm inputs, recent yield of the varieties grown, postharvest handling, and marketing.

### Designing questionnaires

A structured questionnaire was used to collect data for this study. The designing of the questionnaires took several factors into consideration [18]. The questionnaires were developed based on the study’s objectives, ensuring that they included the information needed to answer questions that support achieving those objectives. Further to this, the questionnaires were designed by formulating structured questions that were simple to understand and avoiding bias. The questions were also formulated with consideration of the target population in a manner that the target respondents would easily understand. The questions were organised in a sequence to avoid confusion when presented to the respondents. The questionnaires were formulated in a way that conforms to the format which the information collected was to be easily analysed. The questionnaires were pre-tested by presenting questions to some selected households in the target area. Two questionnaires were developed; one specifically targeting the formal seed system and the other targeting the informal seed system.

### Data analysis

Data was analysed using SPSS Version 23 software [19], for all the components reported.

#### Statistical Treatment

Probability sampling was applied to collect household data on the following parameters: demographic data including farmers’ sex, age, household size (number of members for each household), literacy levels (number of years spent in school), and major sources of information for the farmers; proportion of seed production by local seed companies; descriptive information on agrodealers; sources of information for agrodealers; proportion of extension services provided by agrodealers; availability and access of germplasm of maize and groundnuts by different chain actors; reliability of seed suppliers; proportion of promotion activities for popularizing new varieties; key sources of germplasm; data on key research institutions, regulatory authorities and companies involved in the VC.

## Results and discussion

### Actors in the seed value chain for maize and groundnut

**Fig. 2:**
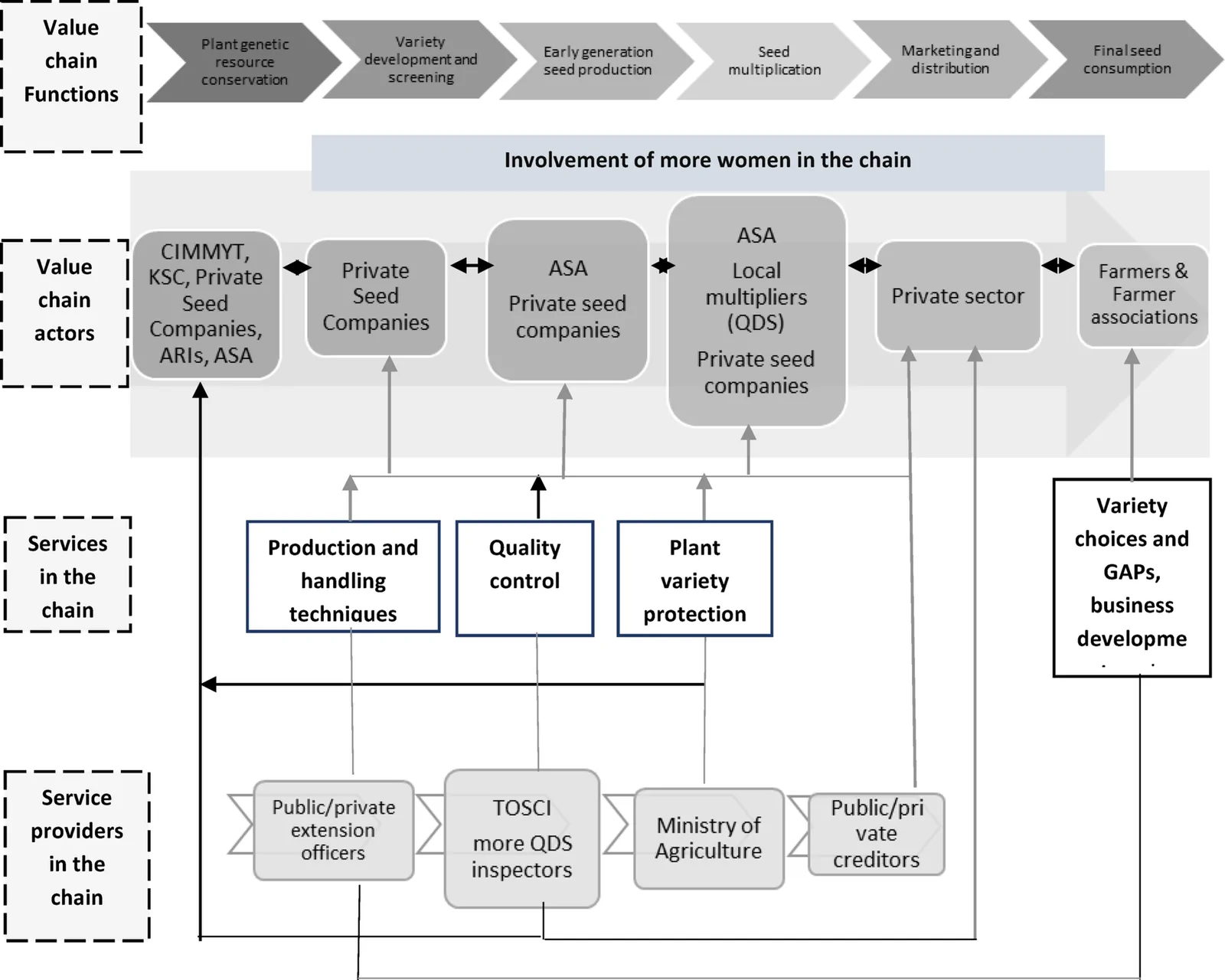
Suggested framework for a well-designed seed value chain for maize in Northern and Central Tanzania

**Fig. 3:**
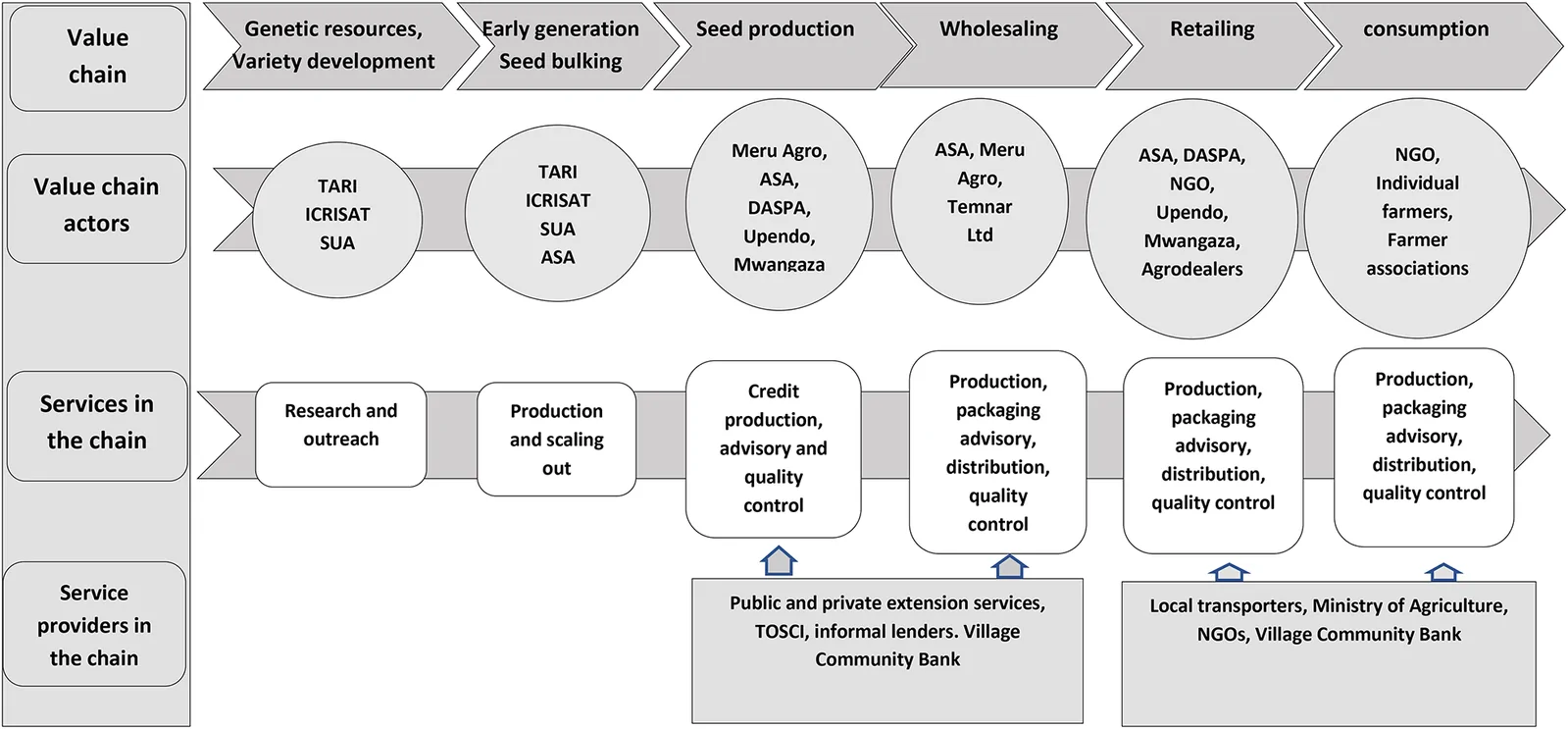
Suggested framework for a well-designed seed value chain for groundnut in Central Tanzania

Informed by contributions from the value chain actors, the study resulted in the development of proposed seed value chain maps as a model, for maize and groundnut, outlining a well-designed seed value chain depicting well-coordinated processes implemented by stakeholders that support the seed systems and promoting private sector investment opportunities in central and northern Tanzania (Figs. 2 and 3).

The proposed models suggest that the seed business and delivery system require a well-coordinated effort among stakeholders; from research institutions on variety development and production, regulatory institutions on quality and registration of new varieties, seed business entities for seed production, distribution and retail, extension and other sources for information dissemination and promotional activities, and policy environment to provide good operational framework. The roles of the actors are presented in the following subsequent topics and summarized in Table 2.

### Seed producers

#### K-means cluster analysis

K-means cluster analysis [20] performed (Table 3); identified only classification of 3 clusters that had some significant difference at 5% level for; standardized (z-scores) for sex, age, number of years of schooling, number of members living in the household, and main sources of information on improved varieties. Two cluster classification had no significant difference between all the two clusters’ standardized variables. The final cluster centers (Table 4) showed that differences between the standardized household related variables’ clusters were not strong enough to compute statistical differences.

**Table 3:** One-way ANOVA for cluster differences for seed producing households

Variable	Cluster		Error		F	Probability^†^
	Mean Square (Zscore)	Degrees of freedom	Mean Square (Zscore)	Degrees of freedom		
Sex of the farmer	3.424	2	0.448	8	7.636	0.01
Age of the farmer	3.794	2	0.369	8	10.294	0.01
Number of years of schooling	0.552	2	0.380	8	1.455	0.29
Number of members living in this household	0.731	2	0.270	8	2.706	0.13
Main source of this information	3.309	2	0.423	8	7.830	0.01

^†^Significant at *P* < 0.05

**Table 4:** Final Cluster Centers for seed producing households

Variable	Cluster		
	1	2	3
Sex of the maize farmer	− 0.64221	− 0.64221	0.99803
Age of the maize farmer	− 0.96204	0.98757	− 0.49673
Number of years of schooling	0.93465	0.22307	0.22307
Number of members living in this household	− 0.66890	0.20582	− 0.41163
Main source of this information	− 0.85455	− 0.35098	0.99189

#### Composition of maize and groundnut producing households

Farmers are the most critical players in the value chain as they form the end-users of the chain’s products (improved maize and groundnut varieties). Their engagement allows understanding of how seed value chain constraints limit their efforts to adopt improved varieties in central Tanzania, thus affecting the effective demand for seed of these varieties. The demographic characteristics of these households and their responses are summarized in Table 5.

The maize producing households were characterized as follows: 71% of the respondents were men, who were the primary decision-makers in maize farming matters as reported during the focused group discussion. The average age, household size, total farm size, and land under maize varieties were 45 years, 7 persons, 2.2 hectares, and 1.4 hectares, respectively.

The results revealed that 69% of groundnut seed-producing farmers are women while 31% are men (Table 5). This implies that women dominate groundnut seed production in the study area. High involvement of women could be because groundnut is traditionally cultivated by women and seed production could be an extension to the enterprise, they are already familiar with. Commercialization of farming enterprises and equipping the farm by introduction of farm equipment such as oxen-drawn plough, shifts power relations in the enterprise and attracts the participation of more men than women [21].

**Table 5:** Household demographic characteristics of the sample

Demographic Variables	Categories	Maize (*N* = 252)		Groundnut (N = 162)	
		Mean	SD	Mean	SD
Sex	Male (%)	71.0	23.50	31.1	23.30
	Female (%)	29.0	9.70	68.8	25.70
Age	Average	45.0	14.2	46.8	12.60
	Youth (less than 35) (%)	17.6	9.52	20.3	9.60
	Adult (36–55)	57.8	6.95	62.1	6.98
	Elder (Above 55)	20.3	9.50	21.9	9.50
Household size	≤ 6 Members	6.8	3.2	5.0	5.80
Formal education	Number of years	7.1	1.9	7.5	2.1

The average age of groundnut seed producers was 46.8 years, ranging from 20 years to 74 years, consistent with findings in a similar study conducted in Myanmar [22]. This shows that seed producers are old enough and have experience in groundnut production. However, the results showed that 42% of the seed growers were youth (18–35 years) implying that there are good incentives attracting the youth’s participation. The study revealed that an average household had a size of 5 members. Therefore, 70% of the interviewed households registered a household size of no more than 5 members. Focus group discussions revealed that the available family members provided family labour to the households as seed production and agriculture enterprises. Other studies have reported using family labour in farming operations, though over reliance leads to inefficiencies from lack of commitment and obligation [23]. The results in Table 5 indicated that the average number of years of the farmers who attended formal education was 7 to 7.5 for both maize and groundnut, implying that they had basic education, which would enable them to read and write. Exposure to some level of formal education is important providing good potential for communication and understanding the seed business considering that seed enterprise requires good networking and coordination with other actors in the seed VC.

The mean farm size owned by groundnut seed growers was 3.3 hectares. The area under groundnut was 0.8 ha (24.2%) of the total owned land. The results showed that despite the groundnut seed farmers owning big farming land, they allocate a small portion for groundnut seed production, demonstrating that groundnut seed farmers allocate more land to produce other crops with maize having a higher proportion of more than 60% of the owned land.

#### Information sources for farmers and its dissemination among different value chain actors

The study also established the flow of information among different actors of the seed VC and the sources of information for the maize and groundnut seed-producing households. Results of sources of information to the seed-producing households are presented in Table 6.

**Table 6:** Farmers’ main sources of information on improved maize and groundnut varieties

Source	Source of information (%)	
	Maize (N = 252)	Groundnut (N = 162)
NGOs	1.98	-
Seed buyers	1.98	-
Traders	1.98	5.08
Others	3.97	9.30
Media	11.51	14.80
Agrodealers	24.21	5.08
Other farmers	24.21	12.66
Government agricultural extension officers	30.16	58.30

The three main sources of information on improved variety types were extension agents, agrodealers and other farmers (Table 6). The main information source for maize is government agricultural extension officers (30%), followed by other farmers and agrodealers (24%). On average, 16% report getting information from the media (radio, TV, newspapers etc.), while NGOs, seed buyers, and traders each account for only 1.98%.

For groundnut, government agricultural extension officers are even a more dominant source (58%), while media (15%) and other farmers (13%) are also critical; however, agrodealers (5%) are less important as compared to maize, because not many agro-dealers stock groundnut seed.

#### Participation of Commercial Seed Producers and Suppliers

All the owners of local seed companies interviewed as seed producers were men. Largely, few women own agrodealer shops and seed companies. This can be attributed to low access to land and financial resources, and societal bias against women [24, 25]. Financial support to women in the seed supply businesses would encourage more women to be engaged in seed business that could help more farmers access quality seed that could influence increased adoption of improved varieties considering that women in rural areas are engaged in agriculture [24, 26]. The study results showed that, there is little evidence of women involvement in the national and international public Agricultural Research Institutions (ARI) participating in the development and screening of new maize and groundnut varieties. This is a consequence on few women pursuing training in agricultural disciplines therefore, only few are trained in agricultural career fields.

On average, 51.8% of the seed produced by the sampled companies was maize (Table 7), surpassing that of other crops combined. In contrast, the study revealed no well-established seed company dealing with groundnut in central Tanzania. Only two entities operated as seed companies; Dodoma Agricultural Seed Production Association (DASPA) and Muungwano Kilimo. Their main functions were seed production, multiplication, and distribution of seed for various crops and certified seed for groundnut. They all get base stock for seed multiplication from TARI-Naliendele, and they were not actively involved in variety development. These two entities, DASPA and Muungwano are also involved in seed production of other crops, representing 40% and 30% of their business investments, respectively. The two main challenges that both face include poor infrastructure and access to market information. The two seed-producing entities are supported by the research for early generation seed supply and extension department, with extension services supporting pest control, linkage to farmers, market linkage, good farming practices, choice of seed . They both have access to credit at an interest rate ranging from 7% to 18% on the loan facility. DASPA is a member of seed producer’s association that focuses on improving the production of seed, advocacy of common interests in seed production, promotion, and trade fairs or agricultural shows to publicize groundnut, which helps the seed entity secure prior information on effective demand for seed.

**Table 7:** Proportion of seed production by selected local seed companies in Tanzania

Variable	Mean (N = 5)	Std. Deviation	Minimum	Maximum
Percent (%) of maize seed	51.8	41.7	12	95
Percent (%) of other crop seeds	45.8	39.6	5	88
Varieties released	6.3	3.95	3	11
Varieties currently commercialized	4.5	2.4	2	7
Varieties withdrawn	0.3	0.5	0	1

### Business agents

#### Agrodealers in maize and groundnut

Agrodealers are a vital link between farmers and seed supply from the public seed corporations and private companies. They are the retailers in communities and can cover large areas, given their knowledge of both formal and informal seed networks. Table 8 presents some findings of the sampled agrodealers dealing with maize and groundnut seed. Most of the agrodealers (58%) in the study area owned only one outlet. An overwhelming majority of the agrodealers (83.3%) were male. The majority (75%, *N* = 9) of agrodealers sourced their seed of maize from seed companies while the rest acquired them from private distributors. All the agrodealers sold their seeds to smallholder farmers, whereas few of them sold to other buyers including commercial farmers and Non-Governmental Organizations (NGOs). The study revealed that it was a legal requirement to have a business license to be an agrodealer. All agrodealers in the study area sell other agricultural inputs such as fertilizers and pesticides in addition to seeds. The study established that all the agrodealers in the study area operate in their premises.

**Table 8:** Agrodealers profile, their seed source and target customers

Variable	Proportion of agrodealers (%) (N = 12)
Average Number of outlets owned	
1	58.3
2	33.3
4	8.3
Male owned	83.3
Female owned	16.7
*Seed source for Agrodealers*	
Seed companies	75
Distributors	25
*Target customers*	
Smallholder farmers	100
Commercial farmers	66.7
NGOs	41.7
Cooperatives	25
Other agrodealers	8.3

The agrodealers stock seed of different varieties based on required traits that are required such as drought tolerance, early maturity, and shelf life. Physical characteristics such as colour and size were indicated as important market traits for fast sales. The seed supply for groundnut in the region comes from several sources, including, community seed banks, NGOs, and research institutes (TARI and ICRISAT) dominating; with moderate supply from the agrodealers. The agrodealers’ supply was low because there was no major private seed company distributing groundnut seed in the country. This was stated by the agrodealers as a major drawback since the demand for quality seed is high. The other seed suppliers combine seed business with supplying of other inputs such as fertilizers, herbicides, insecticides, pesticides and storage materials such as hermetic bags.

#### Factors that inform agrodealers to stock seed of various varieties

Most agrodealers (34.3–50%) stock a variety preferred by farmers and on demand. The agrodealers incentive is profit maximization, and as such, they would stock farmers’ favourite varieties from any company, regardless of improved varieties from other seed companies. This is further influenced by the ease of access to the variety (20 to 29.2%) from seed companies or wholesalers if they get it at a reasonable price to realize profits (14.3 to 20.8%). This implies that their business is demand-driven, and profit maximization may be their focus through selling products that offer good profits.

#### Sources of information for Agrodealers

**Fig. 4:**
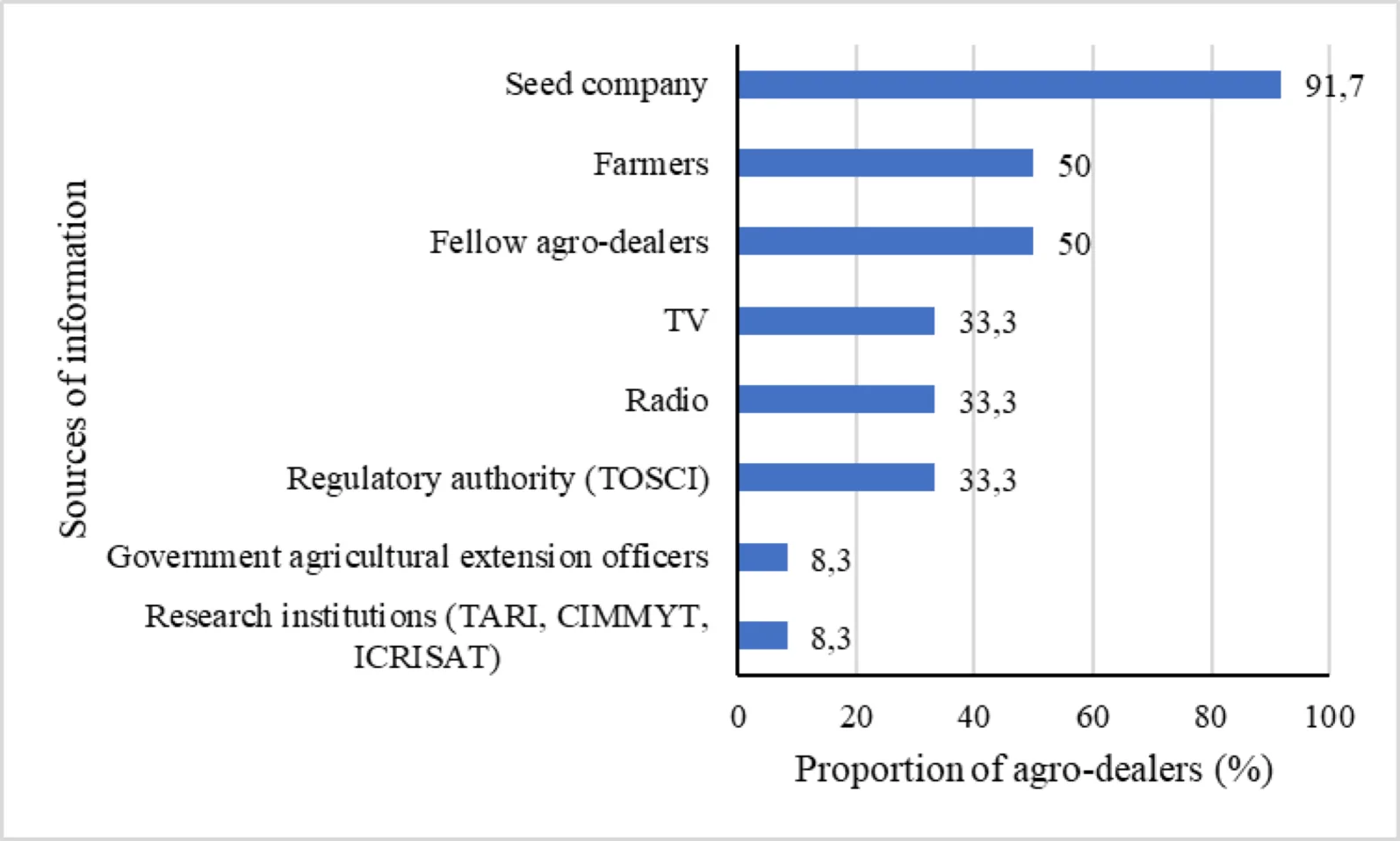
Agro-dealers’ main sources of information on improved maize and groundnut varieties

The sources for information for agrodealers on issues related to the availability of seed of improved maize and groundnut varieties are presented in Fig. 4. Most of the information agrodealers obtained about improved maize and groundnut varieties came from seed companies (92%). Social networks through fellow agrodealers and farmers were also important sources of information on improved maize and groundnut seeds (50%). The use of media including TV and radio was found to be crucial in the provision of advice on agronomic practices such as spacing and variety suitability for specific AEZs. Interestingly, only 8.3% of the agrodealers obtained information from research institutions (e.g., TARI, CIMMYT, ICRISAT) and government agricultural extension officers.

#### Challenges faced by agrodealers

The Agrodealers stated that they face various internal and external challenges in their seed selling businesses (Table 9). The most important internal challenge (41.8%) was inadequate cash to buy stock, since most agrodealers were either micro or small enterprises with limited access to credit. The second most important (33.3%) internal challenge was inadequate seed stock to satisfy farmers’ demand on time. The third, fourth and fifth internal challenges, each accounting for 8.3%, included failure to meet farmers’ seed demand, inadequate storage space, and lack of enough working capital for hiring enough staff.

**Table 9:** Internal and external challenges faced by agrodealers

Challenges	Proportion (%) (N = 12)
*Internal challenges*	
Inadequate cash to buy stock	41.8
Inadequate seed stock to satisfy farmers’ demand in time	33.3
Failure to timely meet farmers’ seed demand	8.3
Inadequate storage	8.3
Lack of enough cash to hire enough staff	8.3
*External challenges*	
High competition from non-agrodealers	33.3
High seed price	25.0
Not enough support with credit	29.15
Not enough support with relevant seed information	29.15

The study further identified external challenges affecting business operations of agrodealers. Among them was high competition from non-agrodealers cited by 33% of the agrodealers. Lack of credit facilities to boost their capital and lack of relevant information on seed were cited as the second most important challenges affecting their business.

### Research Institutions

#### Tanzania agricultural research institute (TARI)

Research plays an important role in the development of improved maize and groundnut varieties. All the staff members interviewed at Selian Agricultural Research Institute (SARI), a research center under TARI located in Arusha, were male. They all indicated that they had descriptors of the improved maize varieties they released in the last four years. Only one of the respondents indicated that they consulted farmers before the development of improved varieties. Others consulted their fellow breeders from other research organizations as well as pathologists. They all indicated that they supply less than 2 tons of foundation seed annually. The ministry of agriculture is the main public sector actor in the seed value chain through TARI. TARI works with the ASA on seed production, ensuring that high-quality seeds are available to farmers at an affordable price. The function of ASA includes expanding seed production and distribution networks to facilitate farmers’ access to seed. Furthermore, ASA has a leading role in ensuring increased private sector participation in seed industry development through the establishment of public-private partnerships and/or joint ventures in seed production and distribution. Thirdly, ASA is mandated to engage in demand stimulation activities of certified seed by farmers; and strengthen research capacities for breeding and development of varieties that address farmers’ specific needs and/or demand.

Results in Table 11 indicate that apart from the research institutes, community seed banks played a crucial role in seed production particularly for groundnut since maize is mostly hybrids that are sold, therefore, mainly produced by seed companies. The community seed banks have a proper leadership structure, though they need to undergo substantial leadership training. Both community seed banks work in collaboration with TARI where they receive support in seed production and extension services. The community seed bank has multiple knowledge streams including seed production principles, seed quality, seed business and post-harvest handling of seed. They sell their seed to vendors, fellow farmers in and outside the village.

**Table 10:** Components supported by extension services and proportion of extension services provided by agrodealers

Extension services	Proportion provided by agrodealers (%) (*N* = 12)
Growing maize and groundnut for seeds	9
Varieties suitable for the area, fertilizer amounts/requirements, agronomy	9
Disease control, the suitability of seeds for different areas	18
Planting, crop management of maize seed, use of herbicides/pesticides and other inputs	63
New maize varieties	50
Inputs use	100
Maize marketing	25
Post-harvest handling	50

### Service providers

#### Extension service providers

Access to extension service providers is critical, therefore, the need to improve on the facilitation of their mobility should be placed into high consideration. Mekdim et al., [27] emphasized incentivizing extension agents by increasing transportation facilities/services to ensure mobility. Some seed companies who were interviewed indicated that they incentivise agrodealers by providing them with transport to ease logistics challenges accessing remote areas as they reach farmers to deliver seed and provide extension support.

The components supported by extension services in the value chain are presented in Table 10. Agrodealers and agricultural extension officers were the major players. Most of the extension officers interviewed were from the public service. Public extension officers provided information about market requirements of seed production. They were also involved in the provision of seed production technologies, post-harvest crop management technologies and marketing information. Concerning maize, 50% of the extension officers organized maize farmers in producer groups while a similar proportion did organize farmers within sub-villages. The extension officers taught farmers on optimal use of inputs and information about new varieties. It was noted that the extension officers did not offer much support to farmers on seed and grain business development, which is crucial for maize and groundnut seed enterprises, and ultimately food security and economic viability. Despite the emphasis on quality seed, optimal inputs use, and good crop management among other good agricultural practices (GAPs), none of the extension agents extended knowledge on credit access to farmers, therefore, lack of access to financial services/credit was identified as a major setback to seed-producing farmers for both maize and groundnut in the study area. On the other hand, all the seed companies interviewed and agrodealers mentioned that they provide free extension services to the farmers they serve. All agrodealers offered extension services on planting, crop management, use of herbicides, and pesticides among other services.

**Table 11:** Source of supply of germplasm and seed, and proportion accessed by VC actors from each supply source

Germplasm and seed supply	Supply source	Proportion (%) of germplasm or seed accessed by chain actors from each source
**Maize**		
*Seed companies’ germplasm sources (N = 5)*		
	ASA	40
	CIMMYT	100
	AATF	20
*TARIs’ germplasm sources (N = 4)*		
	ASA	40
	CIMMYT	100
	AATF	20
	Own collection	20
**Groundnut**		
*TARIs’ germplasm sources (N = 4)*		25
	CGIAR	100
	Own collection	100
	Regional gene banks	50
*Farmer’s ability to access quality seed (N = 252); (N = 162)*		48.4
**Maize (***N* = 252)	Own production	20
	Agrodealer	75
**Groundnut (***N* = 162)	Seed supplier/Agrodealer	10.9
	Community Seed Bank	20.3
	Seed Company	1.6
	NGOs	23.4
	Government projects	14

### Provision of technical support

The study sought to explore who provides technical support for maintaining the germplasm of released improved varieties and developing new varieties (Table 11). The results revealed that maize seed companies acquired their germplasm from CIMMYT, while groundnut seed producers acquired early generation seed from TARI-Naliendele and ICRISAT. The ASA, the only government agency, is also a major player in providing germplasm and early generation seed to seed companies. Seed companies suggested that ASA should quit marketing seeds of improved maize hybrids and focus on producing seed of OPVs and seed of crops such as sorghum and groundnuts, which attract less interest from seed companies. The interviewed seed companies and the QDS inspector pointed out that if ASA was significantly facilitated, there would be a drastic upwards shift in the availability of seed of open-pollinated varieties (OPVs) for cereals and seed of legumes.

**Fig. 5:**
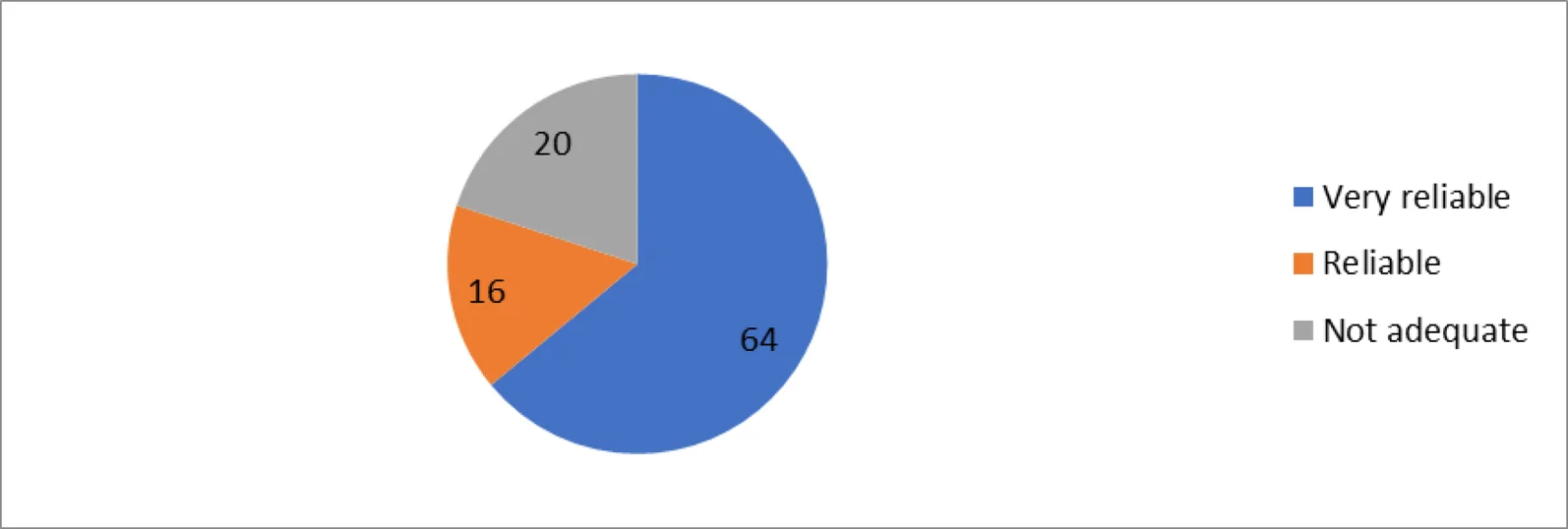
Proportion (%) of farmers indicating reliability of seed suppliers in the study area

On average, 80% of farmers interviewed indicated that groundnut seed suppliers had some degree of reliability, with 64% of respondents indicating that they were very reliable. About 20% of the interviewed farmers indicated that seed suppliers were not reliable (Fig. 5).

### Quality seed promotion activities

The study revealed that different chain actors use diverse promotion activities amidst the heightening competition to enhance farmers’ awareness of improved varieties. These include field demonstrations, field days, training for farmers, agents, free samples, particularly for maize and partnerships with agrodealers. Giving tips to shopkeepers and facilitating the agrodealers with means of transport such as providing a pickup truck seemed to work well for some companies selling maize seed as it was reported to drastically increase the sales of the companies’ varieties. More efforts into the promotion of improved maize seed were evident compared to groundnut, probably due to the more sales from maize seed. The Agrodealers were engaged in promoting new improved maize varieties (Table 12), as opposed to groundnut where fielddemonstrations and field days were key.

**Table 12:** Different promotion activities for new varieties used by agrodealers

Promotion activity	Proportion (%) of options used for promotion (*N* = 12)
Workshops and pieces of training	16.7
Field Demonstration plots	33.3
Display packs	33.3
Free seed samples	58.3
Discounts to farmers	58.3
Recommendation to customers	75.0
Posters	91.7
Others	16.7

The majority (92%) of the agrodealers used posters from the seed companies to promote new maize varieties. On average 75% promoted varieties by recommending them to farmers. Besides the seed companies and agrodealers, TARI promotes improved maize and groundnut varieties. All the sampled TARI staff mentioned that seed fairs, agricultural shows, and demonstration plots were the major channels TARI used to promote new varieties. Most companies suggested that more new crop varieties would penetrate dry areas if different stakeholders involved form strong collaborations and partnerships. However, some companies reported that currently, TARI has concentrated its promotion efforts and partnerships in high-potential areas such as Arusha and Mbeya, thus leaving the high-risk areas such as Kongwa and Kiteto without much attention.

### Policy issues affecting the seed sector

The law provides for compulsory seed certification, laboratory seed testing, variety evaluation and registration under the control of Tanzania Official Seed Certification Institute (TOSCI). The institute is also responsible for testing and National Performance Trials (NPT), which are necessary to facilitate variety release and registration. This is to ensure that quality standards are adhered to as a variety is channelled towards commercialization. The study established that there were delays in issuing stickers of certified seed by TOSCI consequently failing to meet the timeliness of planting and this leads to challenges in the high-risk areas where rainfall is usually limited. A policy gap existed in terms of TOSCI speed of service delivery. The seed producers further reported that the process of variety release was unnecessarily long, which delays the benefits farmers derive from new varieties. As such, this calls for engagement with policymakers for some revision.

Regarding seed pricing, the agrodealers indicated that a policy should be put in place to cap seed prices in the consumer market. They mentioned that some agrodealers even doubled the price of maize seed, whereby groundnut is not an exception, thus hindering the affordability by resource-poor farmers. Some remote areas in the high-risk areas, such as Kiteto and Kongwa, were hit by high prices of seed of improved varieties. However, it is recommended that the price of seed should be driven by demand and supply or the market situations considering that in addition to seed production costs, operational or logistic costs to reach certain markets can be high and vary.

### Challenges and opportunities to improve the groundnut and maize seed value chains in central Tanzania

Seed companies faced seed production challenges such as difficulty in accessing land for seed multiplication. Production of certified seed requires good isolation of the seed production fields from other fields of the same crop, therefore, availability of adequate land is critical for accomplishing this field work to avoid contamination. Seed companies need to increase their investment in land acquisition in Tanzania to meet this requirement. Climate change has resulted in many negative effects including low rainfall, consequently making seed production a great challenge for all companies; leading to the need for irrigation and adoption of more resilient varieties. In addition, the seed companies lacked enough contract farmers as out-growers because most farmers in the region are resource-constrained smallholders. In addition, the available out-growers lack financial resources, compelling seed companies to engage in almost all activities despite the contractual agreements. Therefore, there is need for more opportunities of financial support through microfinancing of agricultural enterprises. Lastly, side marketing, poor seed quality due to poor isolation, and the contamination of improved varieties with other varieties are other major problems that the seed companies face when engaging out-growers in seed multiplication of improved crop varieties.

Cash flow was a major impediment to agrodealers buying stock and limited mobility. Other constraints included inadequate seed supply to satisfy farmers’ demand in time,, inadequate storage, and lack of enough cash to hire enough staff. All these constraints contribute to hinderance on the penetration of improved varieties of groundnut and drought-tolerant maize including Pro-Vitamin A and Quality Protein Maize (QPM), to more areas.

Extension officers faced challenges in their line of duty. For instance, all the extension officers reported that farmers’ low level of education was a major challenge when they trained them on input use. Moreover, extension education on postharvest practices was highly affected by the limited number of input suppliers of postharvest supplies, such as modern storage technology using hermetic bags. Farmers’ failure to accept participation in forming farmer organizations affected their training on good marketing practices. A major drawback to the execution of extension support to farmers originates from the government’s lack of facilitation in the mobility of extension agents.

The efficiency in registration and certification of new varieties was adversely affected by understaffing and under-resourcing by the government, while the private seed companies struggle to have timely access to the basic seed they need from ASA. The over-regulation of the seed sector was reported as complicating the provision of direct licensing agreements between ARIs and private seed companies in bulking foundation seed. Lack of well-established protocols and procedures for maintenance of released public varieties in public agricultural research institutions was reported as contributing to loss of Distinctiveness Uniformity and Stability (DUS) of these varieties.

#### Comparison and opportunities between groundnut and maize seed value chains: Opportunities and prospects

The results from the study revealed that the groundnut seed value chain is under-invested by the private sector including the government seed agency (ASA) compared to maize, although in central Tanzania the case is similar for both. As such, the informal seed systems are predominant in the provision of groundnut seed, with seed supply mostly being done by farmer groups, managed through associations. The groundnut and maize seed value chain are prone to production risks, particularly unfavourable weather and resource constraints to the outgrowers that hamper investment in central Tanzania. The formal seed sector is still weak, with very few private seed companies engaged in production, along with their agrodealer networks to sell groundnut seed, as opposed to the maize seed value chain. However, the private sector shows a slowly growing demand for seed of improved groundnut varieties, mostly driven by the grain market, which supplies grain to the region and country.

The seed production regulatory services are offered at a fee whereby seed companies are mandated to pay in case of producing certified seed, whereas for QDS the fees are paid by the government. The results indicate an opportunity to develop a sustainable seed system for groundnut and drought-tolerant maize OPVs by engaging with stakeholders such as community-based seed producers/seed associations with a strong foundation in central Tanzania, agrodealers and the smaller emerging seed companies.

Strengthening linkages among different actors (Figs. 1 and 2) in both seed value chains to stimulate the market for groundnut and maize to meet farmers’ demand for quality seed is critical. This has shown that even though the public sector supports seed production, neither them nor the private sector are fully involved in ensuring efficient delivery of seed of groundnut and maize OPVs. As such, an integrated seed supply system would offer a sustainable solution, which calls for concerted efforts to have strategic partnerships to achieve this goal. Of importance is to exploit the power of innovation platforms that would help bring different stakeholders together. Generally, improvement of seed systems for the groundnut and maize OPVs including provitamin-A or QPM, requires building the seed value chains from the supply side. The informal seed system would serve as a foundation for the private sector investment in the case of the groundnut seed value chain. The study identified key entry points that would act as economic drivers to farmers in central Tanzania. Seed production was identified as the most feasible option. This can go a long way in stimulating the supply of quality seeds in the region. As such, developing a concrete strategy that would ensure access to basic seed, credit services and extension support to feed the seed production initiative would make the groundnut and maize OPVs seed value chains more vibrant.

## Conclusion

In Tanzania, several factors hinder the competitiveness of maize and groundnut seed value chains. This study conducted in three districts; Babati, Kongwa, and Kiteto, examined the challenges faced by both value chains and explored potential solutions based on lessons learned from a formal seed system compared to the informal seed system of the value chains.

The study established that there are several challenges that affect the value chain actors. Seed companies face challenges like land scarcity, climate impacts, and difficulty securing out-growers due to farmer resource constraints, leading to quality issues. Agrodealers struggle with cash flow, inadequate stock, and storage, hindering the distribution of vital varieties. Extension officers contend with farmers’ low education, limited modern inputs, and poor mobility, impacting training and outreach. Regulatory bodies and research institutions are hampered by understaffing, slow certification, and restrictive policies, which impede private sector access to basic seed and affect production of certified seed of of released varieties.

Seed companies tend to be cautious about producing certified seed in drier districts like Kongwa and Kiteto, likely due to the higher risks associated with limited rainfall. In contrast, Babati district, which benefits from two rainy seasons each year, was seen as a more favorable location for seed production, encouraging more activity from seed producers there. When comparing the maize and groundnut seed value chains, it became clear that most stakeholders including farmers, seed companies, and agrodealers, chose to invest more heavily in maize. This preference was largely driven by the perception that maize offers greater financial returns than groundnut, making it a more attractive option across the value chain.

The results also suggest that most of the seed companies did not have incentives to invest in improved varieties that are drought tolerant because of the dismal performance of these varieties in the dry areas and lack of clear effective demand. The major marketer of OPVs of maize and legumes is ASA; however, the volume of seed produced does not meet the demand for seed. Groundnut and drought tolerant maize hybrids, being drought-resilient, offer an opportunity for the seed companies to invest in seed production of these improved varieties in central Tanzania that has semi-Arid conditions.

The study highlights a real opportunity to build a sustainable seed system for groundnut and drought-tolerant maize in central Tanzania by working closely with community-based seed producers, agrodealers, and emerging seed companies. Stronger connections between these groups are essential to increase the market and ensure farmers have reliable access to quality seeds. While both public and private sectors play a role, neither is fully meeting the needs for seed production of groundnut and also having specialized skills of seed production of some unique types of maize such as pro-vitamin A or QPM. An integrated approach; bringing together all stakeholders through innovation platforms and strategic partnerships; could make a big difference. Strengthening the informal seed system, especially for groundnut, can lay the groundwork for private investment. The study points to seed multiplication as a practical entry point, which, if supported by better access to basic seed, credit, and extension services, could catalyse the entire value chain and improve farmers’ livelihoods in the region.

Future studies can build on the outcomes of this study and generate more information, especially on the market systems and the different seed systems, because these two components have greater influence on the demand and supply of seed.

## Data Availability

The data supporting the results of this article are included within the article; Data are available from CIMMYT and ICRISAT on reasonable requests following standard processes of the institution.
